# Case of Rupture of the Right Auricle of the Heart

**Published:** 1825-03

**Authors:** W. Thomas

**Affiliations:** Member of the Royal College of Surgeons, and Licentiate of the Worshipful Company of Apothecaries, London.


ART. VII.—*Case of Rupture of the Right Auricle of the Heart.* By W. THOMAS, Esq. Member of the Royal College of Surgeons, and Licentiate of the Worshipful Company of Apothecaries, London.

E. H., a lad, fourteen years old, had complained from childhood of severe dyspnœa and great palpitation, increased by the least exertion, from which I was latterly led to suspect an organic disease of the heart,—either ossification of its valves, or aneurism of one of the primitive vessels: the latter appearing rather obscure, from there being no perceptible swelling in any part of the chest. His heart would sometimes beat in such a manner, (there was continually more throbbing than natural,) as to be perceptible through his clothes, even with his coat buttoned. A sense of straightness in the chest, with a dull sensation, rather than pain, at the scrobiculus cordis, would occasionally ensue. Mostly a bluish tint of the lips, slight dilatation of the pupils; appetite and general health good; grew in the ordinary manner; bowels regular; pulse small, quick, and intermitting, and *not corresponding with the throbbing of the heart,* but the same at both wrists.

In September last, I warned the parents of a sudden death; and, on the 11th of January, 1825, while exerting himself by walking pretty smartly, he called his companion to feel his heart, when he instantly dropped down, and expired without uttering a groan.

In twenty-four hours after death, I was allowed one hour to examine the chest. On opening the pericardium, I was remarkably struck with the dimensions of the heart, being nearly double the natural size. In this cavity there was a quantity of dark-coloured blood. The principal vessels leading from the heart much enlarged. When the heart was removed from the body, I found a small, irregular opening (evidently a rupture) leading out of the right auricle, about three-eighths of an inch long, through which the dark-coloured blood had escaped, and which was no doubt the cause of death. The sides of this auricle appeared quite flabby, and easily gave way to pressure under the fingers. The ventricles were healthy, although their parietes were rather thicker than natural. None of the valves ossified. The foramen ovale closed, and the coronary veins loaded with black blood. The lungs appeared quite healthy.

*Melville Town, near Pembroke;*

*29th January,* 1825.

